# Bioprocessing of Damaged Wheat Grain Into PHB Using *Lactobacillus* Fermentation Following Optimized Acid Hydrolysis

**DOI:** 10.1002/fsn3.72491

**Published:** 2026-10-31

**Authors:** Zohreh Boorboormoradi, Orang Eyvazzade, Ladan Rashidi, Leila Nateghi

**Affiliations:** ^1^ Department of Food Science and Technology VaP.C., Islamic Azad University Varamin Iran; ^2^ Research Center of Food Technology and Agricultural Products Standard Research Institute (SRI) Karaj Iran

**Keywords:** crystalline structure, nuclear magnetic resonance, polyhydroxybutyrate, thermal stability

## Abstract

This study establishes a sustainable strategy for poly(3‐hydroxybutyrate) (PHB) biosynthesis by utilizing damaged wheat grain (DWG) as an alternative, non‐food agro‐industrial substrate. The integrated approach involves the systematic optimization of HCl‐mediated hydrolysis followed by microbial fermentation using *Lactobacillus* species. Hydrolysis parameters, including time, acid‐to‐sample ratio, HCl concentration, and substrate loading, were evaluated to maximize reducing sugar (RS) release while suppressing the formation of fermentation inhibitors. Optimal nutrient recovery was achieved at 45 min, 20% w/v acid‐to‐sample ratio, 6% w/v HCl, and 20% w/w substrate loading, yielding 336.66 ± 1.14 mg glucose equivalent/g RS and 4.67% ± 0.07% w/w protein. Under these optimized conditions, inhibitory concentrations of acetic acid, formic acid, furfural, and 5‐HMF were effectively restricted to 2.36, 6.95, 0.49, and 1.17 mg/g, respectively. Subsequent fermentation of the refined hydrolysate by a consortium of *Lactobacillus plantarum* , *Lactobacillus murinus* , and *Lactobacillus casei* was conducted. At 30°C with a 6% inoculum level over 96 h, FH12 achieved a maximum PHB titer of 1.85 ± 0.42 g/L. These results demonstrate the potential of converting low‐value agro‐wastes into biopolymers via optimized lactic acid bacteria fermentation, providing a viable pathway for circular bioeconomy applications.

## Graphical abstract

336.66 mg/g RS from DWG hydrolysis at 45 min with 6% HCl. 1.85 g/L PHB produced by FH12 at 30°C, 6%, after 96 h. NMR and FTIR confirmed the authentic chemical structure of PHB. PHB showed thermal stability from 270°C to 340°C with max loss at 350°C. PHB showed UV‐blocking and crystalline structure for packaging use.

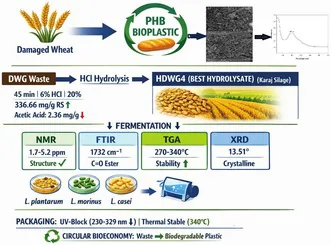

**Abbreviations**
5‐HMF: 5‐hydroxymethylfurfural
DCW: dry cell weight
DNS: 3,5‐dinitrosalicylic acid
DTA: differential thermal analysis
DTG: derivative thermogravimetry
DWG: damaged wheat grain
FESEM: field‐emission scanning electron microscopy
FTIR: Fourier‐transform infrared spectroscopy
HDWG: hydrolyzed damaged wheat grain
HPLC: high‐performance liquid chromatography
NMR: nuclear magnetic resonance
OD: optical density
PHA: polyhydroxyalkanoate
PHB: poly(3‐hydroxybutyrate)
RS: reducing sugars
SD: standard deviation
SEM: scanning electron microscopy
TGA: thermogravimetric analysis
*T*_*m*_
: melting temperature
UV–Vis: ultraviolet–visible spectroscopy
XRD: X‐ray diffraction

## 1 Introduction

Polyhydroxybutyrate (PHB), a member of the polyhydroxyalkanoates (PHA) family, has attracted considerable attention as a biodegradable alternative to petroleum‐based polymers such as polyethylene and polypropylene (Wang et al. 2013; García et al. 2014; Sirohi, Prakash Pandey, et al. 2020; Rashidi 2022; Zhou et al. 2023). PHB is an intracellular microbial polyester composed of repeating 3‐hydroxybutyrate units linked by ester bonds, which gives it mechanical properties comparable to some conventional plastics (García et al. 2014; Rashidi 2022; Zhou et al. 2023). In contrast to petroleum‐based plastics that can persist in the environment for centuries, PHB is biodegradable and can be decomposed by microorganisms in soil, water, or compost into carbon dioxide and water within a relatively short period (Sirohi, Prakash Pandey, et al. 2020; Sirohi, Pandey, et al. 2020; Rashidi 2022). These characteristics make PHB a promising material from both environmental and functional perspectives. However, broader industrial application remains limited mainly because of its high production cost (Sirohi, Prakash Pandey, et al. 2020; Rashidi 2022). One practical strategy to reduce PHB production costs is the use of inexpensive and renewable feedstocks. In this context, agricultural waste has received growing interest because it is abundant, low‐cost, and often underutilized, while its improper disposal can create environmental problems (Khadem et al. 2019; Wang et al. 2021; Niazi Tabar et al. 2025). Converting agricultural by‐products into PHB offers a dual advantage: waste valorization and production of environmentally friendly biodegradable plastics (Dietrich et al. 2017; Wang et al. 2021; Rashidi 2022). Agricultural residues such as wheat straw, bran, husks, and damaged wheat grains (DWG) are rich in lignocellulosic components, mainly cellulose, hemicellulose, and lignin, and can therefore serve as potential carbon sources for microbial fermentation (Sirohi, Prakash Pandey, et al. 2020; Sirohi, Pandey, et al. 2020). Other agricultural residues, including sugarcane bagasse, rice straw, rice husk, brewer's spent grain, corn cobs, and woody biomass, have also been investigated as carbohydrate‐rich substrates for biopolymer production (Sirohi, Prakash Pandey, et al. 2020).

To convert lignocellulosic residues into PHB, the biomass must first be pretreated and hydrolyzed to release fermentable sugars. This process may involve physical, chemical, or biological treatments, depending on the nature of the feedstock and the desired efficiency of sugar release (Zhao et al. 2018; Sirohi and Pandey 2019; Sirohi, Pandey, et al. 2020). Among available approaches, acid hydrolysis has been reported as an effective strategy for converting compromised wheat grains into fermentable substrates, with hydrochloric acid identified as a suitable treatment method in previous studies (Sirohi, Pandey, et al. 2020; Sirohi 2021). However, hydrolysis must be carefully controlled to maximize sugar release while minimizing inhibitory compounds such as furfural, 5‐HMF, and organic acids. The released sugars can then be utilized by PHB‐producing microorganisms during fermentation, followed by polymer recovery from the microbial biomass through extraction and purification steps (García et al. 2014; Pavan et al. 2019; Sirohi, Prakash Pandey, et al. 2020). Using agricultural residues in this way not only lowers reliance on refined carbon sources, but also helps reduce landfill accumulation, open‐field burning, and associated greenhouse gas emissions (Dietrich et al. 2017; Wang et al. 2021; Sirohi, Prakash Pandey, et al. 2020; Sirohi, Pandey, et al. 2020). Although various microorganisms have been explored for PHB biosynthesis, the efficiency of PHB production depends strongly on both the microbial system and the composition of the hydrolysate. *Lactobacillus* species have been investigated for sugar fermentation and bioconversion processes, and their application may offer an alternative route for PHB production from agricultural hydrolysates (Zhao et al. 2018; Sirohi, Prakash Pandey, et al. 2020; Salarieh and Esmaeili 2025). However, studies specifically examining PHB production from damaged wheat grain hydrolysates using a *Lactobacillus*‐based consortium remain limited. Damaged wheat grain is an underutilized lignocellulosic agricultural residue that can serve as a low‐cost and non‐food feedstock for biopolymer production. In this context, the use of a *Lactobacillus* consortium provides a promising bioconversion platform due to metabolic complementarity, division of labor in mixed‐sugar utilization, and enhanced cross‐protection against organic acid and hydrolysate‐derived stresses (Smid and Lacroix 2013; Papadimitriou et al. 2016). Furthermore, the synergistic interaction under acid stress channels surplus carbon and reducing equivalents into polyhydroxybutyrate (PHB) synthesis as an intracellular redox sink, resulting in higher biopolymer yields than single‐strain fermentations (Obruca et al. 2020). Therefore, this study explores the integrated conversion of damaged wheat grain hydrolysate into PHB as a sustainable valorization strategy.

A consortium of lactic acid bacteria was selected to support the bioconversion of damaged wheat grain (DWG) hydrolysate, which contains fermentable carbohydrates together with organic acids and furan derivatives generated during acid hydrolysis (Sirohi, Prakash Pandey, et al. 2020; Sirohi, Pandey, et al. 2020; Sirohi 2021). “A consortium of lactic acid bacteria was selected to support the bioconversion of damaged wheat grain (DWG) hydrolysate, which contains fermentable hexoses and pentoses alongside organic acids and furan derivatives generated during acid hydrolysis (Sirohi, Prakash Pandey, et al. 2020; Sirohi, Pandey, et al. 2020; Sirohi 2021). The consortium comprised *Lactobacillus plantarum* , *Lactobacillus casei* , and *Lactobacillus murinus* . Among lactic acid bacteria, *Lactobacillus plantarum* and *Lactobacillus casei* are well‐documented for their intracellular polyhydroxyalkanoate (PHA/PHB) accumulation capabilities, as well as their broad substrate spectrum enabling the simultaneous utilization of hexose and pentose sugars via the phosphoketolase and glycolytic pathways (Yüksekdağ et al. 2003; Nicolescu et al. 2023). Furthermore, *Lactobacillus plantarum* exhibits notable tolerance to phenolic inhibitors and furfural derivatives frequently present in lignocellulosic and starch hydrolysates (van Kranenburg et al. 1999; Papadimitriou et al. 2016). *Lactobacillus murinus* was incorporated to complement the community through rapid lactic acid fermentation, biofilm/floc‐associated stability, and cross‐protection under severe acidic conditions (Smid and Lacroix 2013). Thus, the combination was tailored to harness complementary sugar uptake, enhance collective tolerance against hydrolysate inhibitors, and facilitate intracellular polymer accumulation under low‐pH growth‐limited conditions.

The use of these species as a mixed culture was intended to provide broader substrate utilization and greater robustness in the complex DWG hydrolysate than might be achieved with a single strain. However, the present study evaluated the consortium as an integrated culture, and therefore the specific contribution of each strain was not separately determined.

The biomass generated during fermentation was subsequently used for PHB recovery because PHB is accumulated intracellularly within microbial cells. Thus, harvesting the fermented biomass was necessary for extracting and purifying the PHB produced from the sugars released from DWG hydrolysate (García et al. 2014; Sirohi, Prakash Pandey, et al. 2020). This approach enabled the direct conversion of the hydrolysate into a value‐added biopolymer and allowed PHB production to be evaluated based on the recovered microbial biomass.

Accordingly, the present study investigated the production of purified PHB from damaged wheat grains through hydrochloric acid hydrolysis followed by fermentation using *Lactobacillus plantarum* , *Lactobacillus murinus* , and *Lactobacillus casei* . After hydrolysis, reducing sugars, protein content, and inhibitor concentrations were evaluated, and the hydrolysate with the most favorable composition was selected for fermentation. The produced PHB was subsequently extracted, purified, quantified, and characterized using DTA, DTG, TGA, UV–Vis spectroscopy, FTIR, NMR, XRD, and FESEM analyses. To the best of our knowledge, the use of these *Lactobacillus* species for PHB production from damaged wheat grain hydrolysates has not been previously reported. Therefore, this study aimed to evaluate the feasibility of this approach as a sustainable waste‐valorization strategy for PHB production.

## 2 Materials and Methods

### 2.1 Raw Materials

Damaged wheat grains (DWG) were obtained from a silage facility in Karaj, Iran. Before processing, the grains were washed with distilled water, air‐dried, and then oven‐dried at 60°C for 18 h. The dried grains were milled using a hammer mill to obtain particles with a size of approximately 500–800 μm, according to Sirohi et al. (2021). The milled material was stored in sealed containers at room temperature until use.

The fermentation microorganisms consisted of *Lactobacillus plantarum* PTCC 1058, *Lactobacillus murinus* PTCC 322, and *Lactobacillus casei* PTCC 1608, which were purchased from the Microorganisms Bank of Isfahan University (Iran). Before the experiments, each strain was maintained separately on de Man–Rogosa–Sharpe (MRS) agar and subsequently cultivated in MRS broth under anaerobic conditions for 24 h. To prepare the bacterial consortium, equal volumes of the three standardized cultures were mixed at a ratio of 1:1:1. The final inoculum was added to the hydrolysate at the concentration specified for each treatment. The inoculum levels evaluated in the fermentation experiments were 3%, 6%, and 9% (v/v). The same strain ratio and culture‐preparation procedure were used for all treatments. The consortium was selected to combine the carbohydrate‐fermenting capacity and acid tolerance of the three lactic acid bacteria and to improve the utilization of the complex damaged‐wheat‐grain hydrolysate. However, the present study evaluated the consortium as a combined culture and did not separately quantify the contribution of each strain.

Analytical‐grade hydrochloric acid (HCl), sodium hydroxide (NaOH), nitric acid (HNO_3_), and hydrogen peroxide (H_2_O_2_) were used for hydrolysis, neutralization, and sample digestion. Chloroform and concentrated sulfuric acid were used during PHB extraction and quantification, respectively. Deionized water was used throughout the experiments. All chemicals were purchased from Aldrich (USA) and were of analytical grade.

### 2.2 Wheat Grain Analysis

#### 2.2.1 Chemical Composition of Wheat Grains

The moisture, carbohydrate, protein, crude fiber, crude fat, and ash contents of damaged wheat grains (DWG) were determined using the applicable Official Methods of Analysis of the Association of Official Analytical Chemists (AOAC 2023). The analytical parameters, methods, and results are summarized in Table 1a.

**TABLE 1a:** Analytical parameters and methods used for the characterization of damaged wheat grains.

Category	Parameter analyzed	Analytical method/instrument	References
Proximate composition	Moisture content	Oven‐drying method	AOAC (2023)
	Carbohydrate content	AOAC‐based proximate analysis	AOAC (2023)
	Protein content	AOAC‐based method	AOAC (2023)
	Crude fiber	AOAC‐based method	AOAC (2023)
	Crude fat	AOAC‐based extraction method	AOAC (2023)
	Ash content	Incineration method	AOAC (2023)
Mineral composition	Fe, Zn, K, Ca, Mn, Cu, Mg, Al, Cs, P, S, and Co	Acid digestion followed by ICP–OES	Harmankaya et al. (2012), with modifications
Heavy metals	Cd, Hg, Ni, and Pb	Acid digestion followed by ICP–OES	Harmankaya et al. (2012), with modifications

#### 2.2.2 Determination of Minerals and Heavy Metals

Mineral and heavy‐metal concentrations in the damaged wheat grains were determined according to the method described by Harmankaya et al. (2012), with minor modifications. Briefly, approximately 0.5 g of dried and ground sample was digested with 5 mL of 65% HNO_3_ and 2 mL of 35% H_2_O_2_ in a reflux digestion system at the boiling temperature until a clear solution was obtained. After cooling, the digest was diluted to a final volume of 20 mL with deionized water and filtered through Whatman No. 42 filter paper. The concentrations of macro‐ and micronutrients (Fe, Zn, K, Ca, Mn, Cu, Mg, Al, Cs, P, S, and Co) and heavy metals (Cd, Hg, Ni, and Pb) were quantified using inductively coupled plasma–optical emission spectroscopy (ICP–OES; Vista Pro Axial, Varian Pty Ltd., Australia). The parameters, analytical methods, and instruments used for the characterization of DWG are summarized in Table 1a.

### 2.3 Hydrolysis of Damaged Wheat Grains

Damaged wheat grain (DWG) was selected as an underutilized agricultural residue because it contains starch and structural carbohydrate fractions that can be converted into fermentable sugars by acid hydrolysis. However, its composition may differ from that of sound wheat and other lignocellulosic residues because grain damage can affect its moisture, protein, mineral, and carbohydrate contents. Consequently, the hydrolysis conditions were selected by considering both the carbohydrate‐rich nature of DWG and the risk of generating fermentation inhibitors during acid treatment. Previous studies have shown that hydrochloric‐acid hydrolysis can enhance the release of reducing sugars from wheat‐based and other agricultural residues. Nevertheless, excessive acid severity may degrade the released sugars and increase the formation of furfural, 5‐hydroxymethylfurfural (5‐HMF), acetic acid, and formic acid (Sirohi and Pandey 2019; Sirohi, Prakash Pandey, et al. 2020; Sirohi, Pandey, et al. 2020; Sirohi 2021).

To evaluate the effects of hydrolysis conditions, three hydrochloric acid concentrations (2%, 6%, and 10% w/v), three acid‐to‐sample ratios (10%, 15%, and 20% v/w), three substrate loadings (10%, 20%, and 30% w/w), and four hydrolysis times (15, 30, 45, and 60 min) were examined. Hydrolysis was performed using hydrochloric acid (HCl) at 60°C. Following the specified hydrolysis time, each sample was immediately neutralized with 1 N NaOH and centrifuged at 3000 × g for 10 min at 25°C. The resulting supernatants were collected for the determination of reducing sugars, protein, and inhibitor concentrations. All experiments were performed in triplicate, and the results are expressed as mean ± standard deviation.

### 2.4 Analysis of DWG Hydrolysates

#### 2.4.1 Estimation of Reducing Sugars

The reducing sugar (RS) concentration in the hydrolysates was determined using the 3,5‐dinitrosalicylic acid (DNS) method (Sirohi and Pandey 2019). Briefly, 1 mL of the sample was mixed with 3 mL of DNS reagent, thoroughly vortexed, and heated in a water bath at 90°C for 10 min. The reaction mixtures were immediately cooled to room temperature, and the absorbance was measured at 540 nm using a UV–Vis spectrophotometer (Shimadzu UV‐1800, Shimadzu Corp., Japan). A reagent blank containing 1 mL of distilled water instead of the sample was prepared under the same conditions. Reducing sugar concentration in the hydrolysates was calculated from a glucose standard curve (0.1–1.0 mg mL^−1^). The standard curve equation was *A* = 0.765 RS + 0.101 (*R* ^2^ = 0.997), where A represents the measured absorbance. RS was expressed as mg glucose equivalents g^−1^ dry weight. RS (mg glucose equivalents g^−1^ DW) was calculated from the DNS‐derived concentration (mg/mL), then multiplied by total extract volume and dilution factor, and normalized to the initial dry biomass (g).

#### 2.4.2 Estimation of Protein Content

The protein content was measured based on Lowry's method described by Sirohi and Pandey (2019). Briefly, 1 mL of the sample was mixed with 5 mL of standard reagents, copper‐carbonate‐tartarate complex, and 0.5 mL of 1:1 diluted Folin–Ciocalteu reagent. The obtained solution was vigorously vortexed and then kept at a temperature of 30°C for 30 min for incubation in a dark place to form the heteropoly molybdenum blue complex from the reduction of phosphomolybdotungstate. Bovine serum albumin (BSA) was used to prepare a standard curve with an equation of $A=0.625\times P+0.175;R^{2}=0.999$, where “*A*” indicates the absorbance at 660 nm and “*P*” indicates the equivalent protein concentration in mg/mL. Thus, the protein concentrations were expressed as mg/mL.

#### 2.4.3 Estimation of Inhibitors

Inhibitor compounds, namely acetic acid, formic acid, furfural, and 5‐hydroxymethylfurfural (5‐HMF), were quantified in the hydrolysates according to Sirohi, Prakash Pandey, et al. (2020). Analysis was conducted using a Shimadzu high‐performance liquid chromatography (HPLC) system equipped with a Phenomenex Rezex RPM monosaccharide column. Each sample (10 μL) was injected at a flow rate of 0.6 mL/min. Detection was performed at 254 nm for furfural and 5‐HMF, and at 210 nm for acetic and formic acids using a refractive index detector (RID). Hydrolysates with the lowest inhibitor levels and highest reducing sugar content were chosen for subsequent PHB production via fermentation.

### 2.5 Production of Polyhydroxybutyrate (PHB)

#### 2.5.1 Preparation of *Lactobacillus* Inoculum

*Lactobacillus* species, including *Lactobacillus plantarum* PTCC 1058 ( *L. plantarum* ), *Lactobacillus morinus* PTCC 322 (*L. morinus*), and *Lactobacillus casei* PTCC 1608 ( *L. casei* ), were first activated in De Man–Rogosa–Sharpe (MRS) broth. Subsequently, 1% (v/v) of each culture was inoculated into 10 mL of fresh MRS broth and incubated at 37°C for 48 h. Then, 0.1 mL of inoculum was cultivated on MRS agar at a temperature of 37°C for 48 h. For inoculation, one colony of *Lactobacillus* species was transferred to MRS broth and incubated at 37°C for 24 h under stirring at 200 rpm (Orbital Shaker ES‐20, Biosan, Latvia). This pre‐culture can be used for fermentation with an absorbance of 1.0 at a wavelength of 600 nm (Derabli et al. 2022).

#### 2.5.2 Fermentation Process

The fermentation was conducted using a consortium of the three lactic acid bacteria ( *Lactobacillus plantarum* PTCC 1058, *Lactobacillus murinus* PTCC 322, and *Lactobacillus casei* PTCC 1608) to evaluate their synergistic potential for PHB production. The selected hydrolysate with the highest RS content and lowest inhibitor concentration was used as the substrate. Fermentation was performed in 250 mL Erlenmeyer flasks containing 100 mL of medium. The initial pH was adjusted to 7.0; although lactic acid bacteria typically grow optimally at pH 5.5–6.5, a neutral pH was selected to mitigate acid stress and minimize further acidification during the process, which can inhibit PHB accumulation.

Flasks were inoculated with 3%, 6%, and 9% (v/v) of the bacterial consortium (prepared in MRS broth). The flasks were then incubated at two distinct temperatures: 30°C (optimal growth) and 60°C (thermal stress evaluation) with constant stirring at 200 rpm for durations of 24, 48, 72, 96, and 120 h. A total of 30 experimental conditions (designated FH1 to FH30) were tested. All fermentation experiments were performed in triplicate to ensure reproducibility, and results are reported as mean values ± standard deviation.

#### 2.5.3 Extraction and Purification of PHB

PHB extraction and purification were carried out following the method described by Sirohi et al. (2021). Briefly, bacterial biomass was harvested after fermentation by centrifugation at 8000 × g for 10 min at 25°C and subsequently washed with distilled water. The collected cell pellets were dried at 40°C to constant weight and digested using a 40% sodium hypochlorite solution. The digested biomass was centrifuged at 8000 × g for 30 min at 25°C and sequentially washed with distilled water, acetone, and methanol before being dissolved in 25 mL of chloroform. The chloroform solution was left at room temperature to allow complete evaporation of methanol. Subsequently, 1 mg of this sample was treated with 5 mL of concentrated H_2_SO_4_ and incubated at 100°C for 30 min. The absorbance of the resulting solution was analyzed at 235 nm using a UV–Vis spectrophotometer (Shimadzu UV‐1800, Shimadzu Corp., Japan), with crotonic acid serving as the standard. Fermented hydrolysate samples containing PHB were designated FH1 to FH30. The sample exhibiting the highest PHB content was selected for further analysis.

### 2.6 Characteristics of Synthesized PHB Through Analytical Tools

#### 2.6.1 Nuclear Magnetic Resonance (NMR)

The chemical structure of the PHB extracted under optimal conditions (FH12: 1.85 ± 0.42 g/L) was characterized by ^1^H and ^13^C nuclear magnetic resonance (NMR) spectroscopy, as described by Sirohi et al. (2021). Spectra were recorded at room temperature using a Bruker Avance II 500 MHz spectrometer (Bruker, Rheinstetten, Germany) equipped with a dual ^1^H/^13^C probe for structural characterization. For NMR analysis, approximately 20 mg of purified PHB was dissolved in deuterated chloroform (CDCl_3_) before measurement. Chemical shifts (δ) were expressed in parts per million (ppm) relative to the solvent signal. The analysis was performed to confirm the characteristic structural groups of polyhydroxybutyrate, including methyl (—CH3), methylene (—CH2), and methine (—CH) moieties, by comparison with reported literature data.

#### 2.6.2 Fourier Transform Infrared Spectroscopy (FTIR)

The functional groups of PHB were analyzed using Fourier Transform Infrared spectroscopy (FTIR, Cary 630; Agilent, 13 Santa Clara, CA, USA) (Sirohi 2021). For this purpose, 2 mg of PHB samples were analyzed using Fourier‐transform infrared spectroscopy (FTIR). The polymer was blended with KBr, and spectra were recorded in the range of 4000–400 cm^−1^ with a resolution of 4 cm^−1^.

#### 2.6.3 Thermogravimetric and Differential Thermal Analysis (TGA and DTA)

The selected sample with the highest PHB was analyzed for thermogravimetric analysis (TGA), differential thermal analysis (DTA), and derivative thermogravimetry (DTG) using a thermogravimetric analyzer (Mettler, Toledo, USA) (Sirohi et al. 2021). Briefly, 3 mg of PHB powder was weighed and placed in a platinum crucible. The heating program varied for every different analysis. For thermogravimetric analysis (TGA), samples were heated from 0°C to 800°C at a rate of 10°C/min. Differential thermal analysis (DTA) was performed over the range of 0°C–400°C, and derivative thermogravimetry (DTG) was conducted from 0°C to 700°C, both at the same heating rate. Weight loss (%) and the rate of weight change were obtained and plotted against temperature to evaluate the physicochemical degradation profile of the synthesized PHB.

#### 2.6.4 X‐Ray Diffraction Analysis

The X‐ray diffraction (XRD) pattern of the selected sample with the highest PHB sample was assessed using an XRD analyzer (D8 Advance, Bruker, Germany) (Sirohi et al. 2021). For analysis, the PHB powder was finely ground, spread uniformly on the sample plate, and positioned in the instrument holder. Measurements were carried out at 40 kV and 30 mA using Cu–Kα radiation. Diffraction patterns were recorded over a 2θ range of 5°–45° with a scanning speed of 5°/min.

#### 2.6.5 Scanning Electron Microscopy

The PHB sample with the highest yield was examined using scanning electron microscopy (SEM, Carl Zeiss, Japan), following the procedure described by Sirohi et al. (2021). Approximately 10 mg of PHB was placed on a carbon‐coated tape attached to the SEM grid and pressed into a thin layer. The specimen was exposed to a mercury UV lamp for 5 min to minimize possible microbial contamination on the sample surface before SEM imaging and was subsequently air‐dried before imaging. Surface morphology was examined at magnifications of 10.0 k and 30.0 k×.

#### 2.6.6 Optical Spectroscopy

The PHB‐rich sample was characterized for its optical behavior using a UV–Vis spectrophotometer (UV‐2600, Shimadzu, Kyoto, Japan) following the protocol of Sirohi et al. (2021). This analysis was performed to evaluate potential light‐sensitive properties relevant to packaging applications. For measurement, 2 mg of the polymer was dispersed in chloroform (100%) by vortexing for 30 s, followed by sonication (40 kHz) for 1 min to obtain a transparent solution. The solution was then transferred to a 1 mL quartz cuvette, and spectra were recorded within the 190–1100 nm range. The optical properties were presented as absorbance versus wavelength plots.

### 2.7 Statistical Analysis

All experiments were performed in triplicate, and the results are presented as mean ± standard deviation (SD). Data obtained from the UV–Vis spectrophotometer, HPLC, FTIR, NMR, XRD, TGA/DTG/DTA, and SEM analyses were acquired and processed using the software associated with the corresponding instruments. Statistical analysis was performed using IBM SPSS Statistics software, version 2022. One‐way analysis of variance (ANOVA) was used to evaluate significant differences among treatments, and Duncan's multiple‐range test was applied for mean comparisons. Differences were considered statistically significant at *p* < 0.05.

## 3 Results and Discussion

### 3.1 Characteristics of Damaged Wheat Grain

The proximate composition of DWG is presented in Table 1b. DWG contained 13.86% ± 0.26% moisture, 50.97% ± 0.37% carbohydrates, 0.99% ± 0.09% crude fat, 3.99% ± 0.23% protein, 24.34% ± 0.43% crude fiber, and 5.91% ± 0.21% ash. These results indicate that DWG contains considerable carbohydrate and fiber fractions, which may support its use as an inexpensive substrate for fermentation‐based bioprocesses. Compared with the spoiled wheat grain reported by Sirohi and Pandey (2019), the present DWG sample showed a different proximate profile. Spoiled wheat contained 7.18% ± 0.04% protein and 0.83% ± 0.01% fat on a wet‐weight basis, whereas the present DWG contained 3.99% ± 0.23% protein and 0.99% ± 0.09% crude fat. The lower protein content and the differences in fat, fiber, and ash contents may be associated with variation in the type and severity of grain damage, storage history, moisture content, sample origin, and analytical procedures. For comparison, sound wheat is generally reported to contain approximately 14% protein, 2% fat, and 2.5% minerals, although these values vary according to cultivar, geographical origin, moisture basis, and analytical method (Iqbal et al. 2022). Therefore, DWG should be regarded as a heterogeneous agricultural residue rather than a material with a composition identical to sound or spoiled wheat. Comparisons among studies should also be interpreted cautiously when the results are reported on different moisture bases. Nevertheless, the considerable carbohydrate and fiber fractions observed in the present sample support its potential as a low‐cost feedstock for microbial fermentation and biopolymer production.

**TABLE 1b:** Chemical composition of damaged wheat grain (DWG).

Chemical compositions	Content (%w/w)
Humidity/moisture content	13.86 ± 0.26
Carbohydrate	50.97 ± 0.37
Crude fat	0.99 ± 0.09
Protein	3.99 ± 0.23
Crude fiber	24.34 ± 0.43
Ash	5.91 ± 0.21

Mineral, trace‐element, and potentially toxic‐metal concentrations in DWG were quantified using inductively coupled plasma–optical emission spectroscopy (ICP–OES), and the results are presented in Table 1c. The mineral profile was dominated by K (4153.48 ± 61.46 μg g^−1^), P (3254.78 ± 9.92 μg g^−1^), Mg (1191.18 ± 3.72 μg g^−1^), and Ca (512.91 ± 26.65 μg g^−1^). This distribution is consistent with reports identifying P, K, Ca, and Mg as important mineral constituents of wheat grain, although their concentrations may vary substantially among cultivars and production regions (Khalid et al. 2023). These elements may contribute to the nutritional composition of the hydrolysate. In particular, Mg and P are involved in cellular energy transfer, nucleic‐acid synthesis, and enzymatic regulation in microbial systems. However, the present study did not determine the bioavailability of these elements or their individual effects on the growth of the *Lactobacillus* consortium.

**TABLE 1c:** Mineral and trace element composition of damaged wheat grains determined by ICP‐OES (μg g^−1^ dry weight).

Minerals	Content (μg g^−1^)
Ag	< 0.001
Al	25.99 ± 2.83
As	0.12 ± 0.05
B	< 0.01
Ba	0.06 ± 0.01
Bi	< 0.001
Ca	512.91 ± 26.65
Cd	0.01 ± 0.00
Co	0.10 ± 0.01
Cr	0.58 ± 0.16
Cs	< 0.001
Cu	6.39 ± 0.60
Fe	43.09 ± 1.93
Ga	0.03 ± 0.01
Hg	0.05 ± 0.01
K	4153.48 ± 61.46
Li	< 0.001
Mg	1191.18 ± 3.72
Mn	33.17 ± 2.36
Mo	0.29 ± 0.03
Na	93.37 ± 6.82
Ni	2.36 ± 0.78
Pb	0.17 ± 0.02
P	3254.78 ± 9.92
Pt	< 0.001
S	134.79 ± 7.41
Se	0.25 ± 0.02
Sr	27.37 ± 10.40
Ti	0.86 ± 0.21
Zn	34.10 ± 0.46

*Note:* The concentrations of macro‐ and micro‐elements were consistent with previously reported mineral ranges for wheat grains, confirming the reliability of the ICP‐OES analysis. Values reported as “<” indicate concentrations below the detection limit of the ICP‐OES method.

Among the trace elements, Fe (43.09 ± 1.93 μg g^−1^), Zn (34.10 ± 0.46 μg g^−1^), Mn (33.17 ± 2.36 μg g^−1^), and Cu (6.39 ± 0.60 μg g^−1^) were detected. The Fe concentration was within the approximate range of 28.8–56.5 μg g^−1^ reported for wheat grain (Iqbal et al. 2022). These elements are nutritionally relevant because they can participate in enzymatic and cellular functions. Nevertheless, their actual availability to the microorganisms may be affected by the hydrolysis, neutralization, and fermentation conditions. Accordingly, their presence should be interpreted as a potential nutritional contribution rather than as a demonstrated growth‐promoting effect. The concentrations of Cd (0.01 ± 0.00 μg g^−1^), Hg (0.05 ± 0.01 μg g^−1^), and Pb (0.17 ± 0.02 μg g^−1^) were low, whereas Ni was detected at 2.36 ± 0.78 μg g^−1^. Compared with the spoiled wheat reported by Sirohi and Pandey (2019), the present DWG contained higher levels of K, P, Mg, and Ca, whereas its Fe concentration was lower. The most pronounced difference was observed for Pb: the Pb concentration in DWG was 0.17 ± 0.02 μg g^−1^, compared with 30.77 μg g^−1^ reported for spoiled wheat. These values should be interpreted in relation to the intended application and the applicable regulatory framework. The low concentrations of Pb, Cd, and Hg indicate a relatively low toxic‐metal burden in the analyzed DWG sample. However, these results do not establish absolute safety or confirm complete regulatory compliance. Commission Regulation (EU) 2023/915 provides maximum levels for certain contaminants in foodstuffs, including cereals; nevertheless, its direct application to damaged wheat used as a fermentation feedstock requires consideration of the relevant matrix and intended use. Before industrial scale‐up, the substrate, hydrolysate, fermentation broth, recovered biomass, and purified PHB should be evaluated against the applicable national and international requirements. The transfer and removal of metals during hydrolysis, fermentation, biomass recovery, and PHB purification should also be investigated. Furthermore, techno‐economic and life‐cycle assessments were outside the scope of the present study and would be required to evaluate industrial viability, environmental performance, processing costs, and economic competitiveness.

Overall, the elemental profile of the analyzed DWG indicates a distinct composition characterized by substantial levels of essential minerals and relatively low concentrations of the analyzed potentially toxic metals. This profile supports the potential use of DWG as a fermentation feedstock; however, further process‐specific safety and regulatory assessments are required before industrial application (Sirohi and Pandey 2019; Sirohi, Prakash Pandey, et al. 2020).

### 3.2 Characteristics of DWG‐Hydrolysates

#### 3.2.1 Hydrolysis Conditions and Selection of the Optimal Hydrolysate

A total of 27 treatment combinations, designated HDWG1–HDWG27, were investigated, as detailed in Table 2a. Table 2b summarizes the effects of hydrolysis time, acid‐to‐sample ratio, HCl concentration, and substrate loading on reducing‐sugar (RS) release, protein retention, and inhibitor formation. Among the 27 treatments, HDWG4, prepared using 6% HCl, 20% (w/w) substrate loading, a 20% (v/w) acid‐to‐sample ratio, and 45 min of hydrolysis, was selected as the most suitable hydrolysate for subsequent fermentation. HDWG4 yielded 336.66 ± 1.14 mg glucose equivalents g^−1^ DW of RS and contained 4.67% ± 0.07% (w/w) protein. Although its RS concentration was statistically comparable to that of HDWG6, HDWG4 exhibited a more favorable overall profile, with acetic acid, formic acid, furfural, and 5‐HMF concentrations of 2.36, 6.95, 0.49, and 1.17 mg g^−1^, respectively. Thus, HDWG4 was selected based on the combined consideration of fermentable‐sugar availability, protein retention, and inhibitor formation rather than on RS concentration alone. The release of RS during acid hydrolysis primarily results from the proton‐catalyzed cleavage of glycosidic bonds in starch fractions, including amylose and amylopectin, producing soluble oligosaccharides and glucose (Santos et al. 2018). These reducing sugars served as fermentable carbon and energy sources for the bacterial consortium during the subsequent fermentation step, supporting cellular growth and the metabolic flux required for intracellular PHB accumulation. However, excessive acid severity may promote secondary sugar‐degradation reactions and the formation of phenolic compounds, furfural, 5‐HMF, and organic acids (Sirohi, Pandey, et al. 2020).

**TABLE 2a:** Experimental design and independent variables used for acid hydrolysis of damaged wheat grains (DWG).

Treatments	Time of sampling (min)	Acid‐to‐substrate ratio (% w/v)	Acid concentration (% w/v)	Substrate (% w/w)
HDWG1	30	15	2	10
HDWG2	45	15	6	30
HDWG3	30	10	10	20
HDWG4	45	20	6	20
HDWG5	30	20	6	10
HDWG6	45	15	10	20
HDWG7	45	15	2	20
HDWG8	45	10	6	20
HDWG9	15	15	10	20
HDWG10	30	10	6	10
HDWG11	30	15	6	20
HDWG12	60	15	6	20
HDWG13	30	10	6	30
HDWG14	30	15	10	30
HDWG15	15	20	6	20
HDWG16	45	15	6	10
HDWG17	60	20	6	20
HDWG18	60	15	2	30
HDWG19	15	15	6	10
HDWG20	30	20	10	20
HDWG21	30	15	10	10
HDWG22	15	15	2	20
HDWG23	15	15	6	30
HDWG24	30	20	6	30
HDWG25	30	10	2	20
HDWG26	15	10	6	20
HDWG27	30	20	2	20

**TABLE 2b:** Responses to the effects of independent variables mentioned in Table 2a (hydrolysis of DWG).

Treatments	RS (mg glucose equivalent/g dry weight)	Protein content (%w/w)	Inhibitors (mg/g)			
			Acetic acid	Formic acid	Furfural	5‐HMF
HDWG1	242.96 ± 1.59^i^	4.29 ± 0.07^d^	5.28	11.75	1.07	2.35
HDWG2	296.30 ± 3.29^b^	4.40 ± 0.06^c^	2.53	8.47	0.74	1.69
HDWG3	221.37 ± 1.64^m^	4.18 ± 0.09^ef^	5.98	13.12	1.21	2.61
HDWG4	336.66 ± 1.14^a^	4.67 ± 0.07^a^	2.36	6.95	0.49	1.17
HDWG5	274.77 ± 1.58^d^	4.38 ± 0.11^cd^	4.27	9.79	0.87	1.95
HDWG6	335.07 ± 2.32^a^	4.59 ± 0.07^a^	2.19	6.05	0.50	1.20
HDWG7	282.22 ± 2.62^c^	4.38 ± 0.07^cd^	4.04	9.32	0.82	1.87
HDWG8	284.76 ± 1.44^c^	4.42 ± 0.12^c^	2.95	9.18	0.81	1.83
HDWG9	189.29 ± 2.39^o^	3.96 ± 0.02^g^	6.96	15.14	1.41	3.02
HDWG10	227.36 ± 2.20^k^	4.09 ± 0.04^f^	5.75	12.73	1.17	2.55
HDWG11	253.90 ± 1.48^h^	4.26 ± 0.02^e^	4.94	11.12	1.00	2.21
HDWG12	256.45 ± 1.81^g^	4.29 ± 0.04^d^	4.86	10.95	0.99	2.18
HDWG13	224.60 ± 0.41^l^	4.10 ± 0.03^f^	5.85	12.90	1.19	2.58
HDWG14	253.12 ± 1.09^h^	4.24 ± 0.05^e^	4.94	11.12	1.01	2.23
HDWG15	180.28 ± 1.24^q^	3.90 ± 0.07^g^	7.23	15.70	1.46	3.13
HDWG16	303.62 ± 2.04^b^	4.48 ± 0.08^b^	2.39	6.97	0.69	1.58
HDWG17	256.84 ± 1.19^g^	4.25 ± 0.08^e^	4.85	10.94	0.99	2.17
HDWG18	237.20 ± 2.07^j^	4.24 ± 0.04^e^	5.45	12.15	1.11	2.43
HDWG19	195.70 ± 0.88^n^	4.02 ± 0.10^fg^	6.76	14.70	1.36	2.94
HDWG20	276.70 ± 1.57^d^	4.36 ± 0.06^d^	4.21	9.68	0.86	1.93
HDWG21	250.71 ± 1.90^h^	4.31 ± 0.02^d^	5.04	11.28	1.03	2.25
HDWG22	181.64 ± 1.81^pq^	3.93 ± 0.06^g^	7.18	15.59	1.45	3.12
HDWG23	183.84 ± 2.33^p^	4.00 ± 0.02^g^	7.12	15.47	1.44	3.09
HDWG24	268.65 ± 1.41^e^	4.38 ± 0.04^cd^	4.47	10.16	0.92	2.03
HDWG25	219.35 ± 2.09^m^	4.07 ± 0.07^f^	6.00	13.24	1.22	2.65
HDWG26	172.85 ± 1.92^r^	3.81 ± 0.02^h^	7.49	16.18	1.51	3.22
HDWG27	263.59 ± 1.41^f^	4.38 ± 0.03^cd^	4.65	10.46	0.94	2.09

*Note:* HDWG4 selected as optimum. HDWG6 statistically comparable in RS. Different lowercase letters indicate significant differences between the columns (*p* < 0.05).

This effect was evident in HDWG26, which exhibited the lowest RS concentration (172.85 mg g^−1^ DW) and protein content (3.81% w/w), together with the highest concentrations of acetic acid (7.49 mg g^−1^), formic acid (16.18 mg g^−1^), furfural (1.51 mg g^−1^), and 5‐HMF (3.22 mg g^−1^). Under severe acid conditions, pentoses may undergo dehydration to form furfural, whereas hexoses may be converted to 5‐HMF. Further degradation of 5‐HMF can generate formic acid and levulinic acid, thereby decreasing the concentration of recoverable fermentable sugars (Derabli et al. 2022; Liu et al. 2012). The results obtained for HDWG26 therefore suggest that excessive hydrolysis severity favored sugar degradation and inhibitor formation rather than efficient sugar recovery.

Acetic acid concentrations across the treatments ranged from 2.19 to 7.49 mg g^−1^. The acetic acid concentration in HDWG4 was 2.36 mg g^−1^, substantially lower than the reported inhibitory range of 8–12 mg g^−1^ for some *Lactobacillus* species under specific experimental conditions (Derabli et al. 2022). This comparison should be interpreted cautiously because microbial inhibition depends on species or strain, pH, temperature, culture medium, and the concentration basis used. Nevertheless, the comparatively low acetic‐acid concentration in HDWG4 indicates a lower potential inhibitor burden for the subsequent fermentation than that observed in HDWG26.

The RS concentration obtained for HDWG4 was higher than the value reported by Sirohi, Pandey, et al. (2020), who obtained 116.29 mg mL^−1^ RS after hydrolysis of DWG with 3% HCl for 45 min. However, this comparison should be interpreted cautiously because the two studies may differ in substrate composition, acid concentration, substrate loading, hydrolysis temperature, solid‐to‐liquid ratio, analytical procedure, and reporting basis. Therefore, the values should not be considered directly equivalent unless the units and dry‐weight basis are first harmonized. Sirohi, Pandey, et al. (2020) nevertheless reported that HCl provided a favorable balance between sugar release and inhibitor formation compared with H_3_PO_4_, H_2_SO_4_, and HNO_3_. The use of HCl may also offer practical advantages because of its established availability and relatively simple handling in dilute‐acid hydrolysis processes.

Derabli et al. (2022) further demonstrated that the optimization of acid‐hydrolysis conditions is important for maximizing fermentable‐sugar recovery while limiting furfural formation during the processing of agro‐industrial residues. They reported sugar recovery of approximately 24 g L^−1^ under optimized conditions using 1% (v/v) HCl at 120°C. The differences between their results and those of the present study may be explained by differences in substrate type, acid concentration, temperature, reaction time, solid loading, and analytical basis. These comparisons support the importance of optimizing hydrolysis conditions for each specific agricultural residue rather than directly transferring conditions from one feedstock to another.

Overall, HDWG4 was selected as the feedstock for subsequent PHB fermentation because it provided a high and statistically favorable RS concentration, adequate protein retention, and comparatively low concentrations of hydrolysis‐derived inhibitors. The hydrolysate therefore supplied potential carbon and nitrogen sources for microbial growth and PHB accumulation. Nevertheless, the present results demonstrate substrate suitability at the laboratory scale and should not be interpreted as conclusive evidence of cost‐effective or industrially sustainable production. Further work involving residual‐sugar analysis, biomass formation, PHB productivity, polymer recovery, detoxification requirements, techno‐economic assessment, and life‐cycle assessment is required to evaluate the feasibility of scale‐up.

### 3.3 PHB Production Through Microbial Fermentation

The PHB‐production potential of the bacterial consortium comprising *Lactobacillus plantarum* PTCC 1058, *Lactobacillus murinus* PTCC 322, and *Lactobacillus casei* PTCC 1608 was evaluated using the selected HDWG4 hydrolysate. Based on the hydrolysis results presented in Table 2b, HDWG4 contained 336.66 ± 1.14 mg glucose equivalents g^−1^ DW of reducing sugars (RS). At a nominal substrate loading of 10% (w/v) in the fermentation medium, this concentration corresponded to approximately 33.6 g L^−1^ of reducing sugars.

The generated reducing sugars served as the principal fermentable carbon and energy source for the bacterial consortium. Following uptake by the cells, glucose and other soluble reducing carbohydrates can be metabolized through glycolysis and related central metabolic pathways, generating pyruvate and acetyl‐CoA. These intermediates may support biomass formation, cellular maintenance, and carbon flux toward intracellular PHB accumulation. Therefore, the suitability of the hydrolysate for PHB production depended not only on the initial RS concentration but also on protein availability, inhibitor concentration, pH, and the ability of the consortium to assimilate the hydrolysate components.

The effects of fermentation temperature, inoculum level, and incubation time on PHB accumulation are summarized in Table 2c. These parameters were evaluated because they affect microbial growth rate, substrate consumption, acid formation, cellular stress, and the balance between biomass synthesis and intracellular polymer storage.

**TABLE 2c:** Effects of incubation temperature (30°C–60°C), inoculum level (3%–9%), and fermentation time (24–120 h) on PHB production (g/L) from HDWG4 hydrolysate by *Lactobacillus* species (Treatment FH12 gave the highest PHB yield).

Treatments	Temperature of incubation (°C)	Inoculum level (%)	Time of incubation (h)	PHB (g/L)
FH1	30	3	96	1.35 ± 0.29^i^
FH2	30	9	96	1.67 ± 0.35^c^
FH3	60	3	24	0.80 ± 0.15^q^
FH4	60	6	72	0.82 ± 0.15^g^
FH5	30	9	120	0.96 ± 0.19^d^
FH6	30	3	120	1.09 ± 0.23^l^
FH7	60	3	96	1.14 ± 0.23^s^
FH8	30	3	72	1.20 ± 0.25^f^
FH9	60	9	24	1.25 ± 0.26^n^
FH10	30	9	48	1.58 ± 0.37^e^
FH11	30	6	72	1.81 ± 0.38^b^
FH12	30	6	96	1.85 ± 0.42^a^
FH13	60	9	48	0.84 ± 0.16^m^
FH14	30	6	120	0.86 ± 0.16^c^
FH15	30	3	48	0.88 ± 0.17^j^
FH16	60	6	24	0.90 ± 0.17^l^
FH17	30	3	24	0.92 ± 0.18^g^
FH18	60	3	72	0.95 ± 0.22^r^
FH19	60	9	96	0.99 ± 0.19^n^
FH20	60	9	72	1.01 ± 0.20^l^
FH21	60	9	120	1.04 ± 0.21^o^
FH22	60	6	96	1.07 ± 0.22^j^
FH23	60	3	120	0.98 ± 0.022^t^
FH24	30	9	24	1.17 ± 0.24^h^
FH25	30	9	72	1.22 ± 0.27^f^
FH26	60	6	120	1.30 ± 0.28^m^
FH27	60	6	48	1.63 ± 0.34^k^
FH28	30	6	48	1.78 ± 0.41^b^
FH29	60	3	48	0.79 ± 0.14^p^
FH30	30	6	24	0.76 ± 0.14^d^

*Note:* Different small letters indicate significantly differences between the columns (*p* < 0.05).

#### 3.3.1 Effect of Fermentation Parameters on PHB Yield

Statistical analysis showed that temperature, inoculum level, and incubation time significantly affected PHB production (*p* < 0.05). The maximum PHB concentration, 1.85 ± 0.42 g L^−1^, was obtained in treatment FH12 at 30°C, 6% inoculum, and 96 h. This was followed by FH11 (1.81 g L^−1^) and FH28 (1.78 g L^−1^). The higher PHB concentration obtained with a 6% inoculum suggests that this level provided a favorable balance between the initial number of metabolically active cells and the available nutrients in the hydrolysate.

The lower inoculum level, represented by FH1, produced 1.35 g L^−1^ PHB, indicating that insufficient initial biomass may have delayed substrate assimilation and polymer accumulation. Conversely, increasing the inoculum to 9%, represented by FH2, resulted in 1.67 g L^−1^, which was lower than the value obtained with the 6% inoculum. This reduction may be related to faster consumption of available nutrients, increased competition among cells, or greater formation of organic acids. However, because residual sugars, biomass concentration, and organic‐acid production were not measured for each treatment, these explanations should be considered plausible interpretations rather than directly verified mechanisms.

The effect of inoculum level observed in the present study is also consistent with the need to balance initial biomass formation and nutrient availability during PHB fermentation. An insufficient inoculum may delay substrate utilization, whereas an excessive inoculum may accelerate nutrient depletion and increase the formation of competing metabolites. Similar dependence on cultivation and nutritional conditions has been reported for PHB production using agro‐industrial substrates (Hamieh et al. 2013; Sirohi et al. 2021).

Temperature had a pronounced effect on PHB accumulation. Increasing the temperature from 30°C to 60°C resulted in an approximately 48%–59% reduction in PHB concentration across the evaluated treatments. The highest PHB concentration observed at 60°C was 1.63 ± 0.34 g L^−1^ in FH27, whereas the lowest value was 0.95 ± 0.22 g L^−1^ in FH18. The superior performance at 30°C is consistent with the mesophilic nature of the evaluated *Lactobacillus* species. Moderate temperature may provide more favorable conditions for cellular growth, sugar assimilation, enzyme activity, and intracellular polymer accumulation. In contrast, elevated temperature may impose physiological stress, alter membrane function, accelerate acid accumulation, or reduce the activity and stability of enzymes associated with PHB biosynthesis.

The predominance of mesophilic conditions in the present study is consistent with previous reports showing that PHB production is highly dependent on the physiological characteristics of the producing microorganism and the cultivation conditions. Sirohi et al. (2021), for example, obtained the highest PHB production from *Bacillus* sp. NII2 at conditions different from those used in the present *Lactobacillus*‐based process. This difference highlights that the optimum temperature and fermentation time cannot be transferred directly between microorganisms or substrates and must be determined experimentally for each production system.

Although some members of the consortium may tolerate higher temperatures, the present results indicate that thermotolerance did not correspond to maximum PHB productivity. The observed temperature dependence also agrees with previous studies showing that PHB production is strongly influenced by the interaction between microbial physiology, substrate composition, and cultivation temperature. Differences in optimum temperature among studies may reflect the microorganism used, the composition of the hydrolysate, pH control, oxygen transfer, and the method used for PHB quantification.

#### 3.3.2 Dynamics of Fermentation Time and Inoculum Level

PHB accumulation increased with fermentation time and reached its maximum after 96 h, followed by a significant decline at 120 h. In the 6% inoculum group, the PHB concentration decreased from 1.85 g L^−1^ at 96 h in FH12 to 0.86 g L^−1^ at 120 h in FH14. This temporal pattern indicates that prolonged cultivation beyond the optimum did not improve polymer accumulation.

The decline after 96 h may be associated with depletion of readily assimilable reducing sugars and subsequent mobilization of intracellular PHB as a carbon and energy reserve. In addition, prolonged fermentation may increase organic‐acid accumulation, alter the pH, reduce cell viability, or promote polymer degradation. The use of intracellular PHB as an alternative carbon source is a biologically plausible explanation; however, it was not directly confirmed in the present study because residual reducing sugars, biomass concentration, pH variation, and PHB turnover were not measured at all fermentation times. Accordingly, the decline should be interpreted as being possibly related to substrate depletion and intracellular polymer mobilization.

The effect of inoculum level further demonstrated the importance of balancing biomass formation and nutrient availability. Increasing the inoculum from 3% to 6% increased PHB production from 1.35 g L^−1^ in FH1 to 1.85 g L^−1^ in FH12. This improvement may reflect faster establishment of the consortium and more efficient utilization of the hydrolysate. However, increasing the inoculum to 9% reduced the PHB concentration to 1.67 g L^−1^ in FH2. A high initial cell density may accelerate sugar consumption and acid production, potentially reducing the carbon available for polymer storage. It may also increase the proportion of carbon directed toward maintenance metabolism or organic‐acid formation rather than PHB accumulation. Since lactic‐acid concentration was not determined for each fermentation treatment, the proposed shift toward lactic‐acid production remains a possible explanation and requires confirmation in future studies.

Overall, the highest PHB accumulation was obtained with a 6% inoculum and 96 h of fermentation, indicating that these conditions provided a favorable balance between microbial growth, reducing‐sugar utilization, and intracellular polymer storage.

#### 3.3.3 Substrate Suitability and Comparative Analysis

The maximum PHB concentration obtained in the present study was 1.85 ± 0.42 g L^−1^, which was higher than the 1.305 g L^−1^ reported by Sirohi et al. (2021) using *Bacillus* sp. NII2 cultivated on enzymatically hydrolyzed damaged wheat grains. In that study, the maximum PHB accumulation reached approximately 34% of the dry cell weight, with a reducing‐sugar concentration of approximately 32.07 g L^−1^ under the optimized conditions. The present study produced a comparable concentration of fermentable sugars in the fermentation medium, approximately 33.6 g L^−1^, but achieved a higher PHB concentration of 1.85 g L^−1^. This difference may be related to the microorganism used, the type of hydrolysis, the composition of the hydrolysate, the inoculum level, the fermentation time, and the PHB extraction and quantification procedures. Therefore, the higher PHB concentration observed in the present study should not be attributed exclusively to the *Lactobacillus* consortium or to the HDWG4 hydrolysis condition.

The use of agro‐industrial residues for PHB production has also been reported for other microorganisms and substrates. Hamieh et al. (2013) reported PHB production by *Lactobacillus acidophilus* using date molasses and whey‐based media. Under their cultivation conditions, approximately 0.412 g PHB per 50 mL of culture was obtained, corresponding to approximately 8.24 g L^−1^. Following statistical optimization of the nutritional factors, a maximum PHB concentration of 43.1 g L^−1^ was reported. However, these values were obtained using supplemented agro‐industrial media and different cultivation conditions and therefore cannot be directly compared with the present DWG hydrolysate system. Nevertheless, the findings of Hamieh et al. (2013) support the potential of *Lactobacillus* species to convert agro‐industrial carbon sources into PHB.

The performance observed in the present study may have resulted from the combined effects of the selected hydrolysate and the optimized fermentation parameters. HDWG4 supplied a high concentration of reducing sugars while maintaining relatively low concentrations of furfural, 5‐HMF, acetic acid, and formic acid. The reducing sugars provided fermentable carbon for microbial growth and PHB biosynthesis, whereas the comparatively low inhibitor concentrations may have reduced the risk of inhibition during fermentation. Sirohi et al. (2021) similarly demonstrated that damaged wheat grain can serve as a low‐cost substrate for PHB production, although their process used enzymatic hydrolysis and *Bacillus* sp. NII2 rather than acid hydrolysis and a *Lactobacillus* consortium.

The present results also demonstrate the importance of optimizing fermentation conditions. The highest PHB concentration was obtained at 30°C, 6% inoculum, and 96 h, whereas higher temperature, excessive inoculum, or prolonged fermentation reduced PHB accumulation. Comparable studies have shown that PHB production depends strongly on the interaction between microorganism physiology, carbon‐source availability, cultivation temperature, inoculum size, and fermentation time (Sirohi et al. 2021; Hamieh et al. 2013). Because these studies used different microorganisms and culture media, the comparison should be considered qualitative rather than a direct ranking of process performance.

The initial pH of 7.0 may have provided a suitable starting condition for the bacterial consortium. However, the pH was not monitored throughout fermentation. Therefore, it cannot be concluded that the initial pH prevented acidification during cultivation. Continuous monitoring of pH, residual reducing sugars, biomass, and lactic‐acid concentration would be necessary to determine the contribution of substrate depletion and organic‐acid accumulation to PHB production.

Overall, the results demonstrate that acid‐hydrolyzed DWG can serve as a promising laboratory‐scale substrate for PHB production by the evaluated *Lactobacillus* consortium. The hydrolysis step generated fermentable sugars that supported microbial growth and intracellular PHB accumulation, while the selected HDWG4 condition limited the formation of major degradation products. However, further investigations are required to assess sugar‐consumption kinetics, biomass yield, PHB productivity, polymer recovery, molecular characteristics, residual inhibitor transfer, techno‐economic feasibility, and environmental performance before considering scale‐up or industrial application.

### 3.4 Characterization of Synthesized PHB

#### 3.4.1 NMR Analysis

The ^1^H‐NMR and ^13^C‐NMR spectra of the selected FH12 sample (1.85 ± 0.42 g/L PHB) are presented in Figure 1, showing strong congruence with PHB spectra reported from *Bacillus* sp. NII2 cultivated on DWG hydrolysate (Sirohi et al. 2021). Characteristic ^1^H‐NMR peaks confirmed the PHB structure (Etxabide et al. 2022), specifically through a prominent resonance at δ1.7–1.9 ppm, which represents the aliphatic protons (methylene —CH_2_— and methyl groups), and a distinct multiplet at δ 5.0–5.2 ppm, corresponding to the methine (—CH—) group at the C—O—C=O position. These detected ^1^H‐NMR signals closely match literature PHB standards (Etxabide et al. 2022; Hassan et al. 2016; Mohapatra et al. 2017; Sirohi et al. 2021). For comparison, *Bacillus* sp. NII2 PHB from DWG showed signals at *δ* 0.86–0.90 ppm (—CH_3_), *δ* 1.30 ppm (—CH_2_—), *δ* 2.47–2.64 ppm (—CH_2_‐ next to ester), and *δ* 5.27 ppm (—CH—) (Sirohi et al. 2021). The ^13^C‐NMR spectrum further validated the PHB structure with major signals at *δ* ≈63ppm (methylene carbon adjacent to the ester), *δ* 71–75 ppm (central methine carbon), and a key resonance at δ≈180ppm representing the quaternary carbonyl carbon (C=O, cf. 169.1 ppm; Sirohi et al. 2021). The characteristic carbon region (60–75 ppm, representing —CH_3_, —CH_2_—, and —CH—) matched literature benchmarks with minor experimental shifts (Mohapatra et al. 2017; Sirohi et al. 2021). These minor chemical shift variations reflect the specific purity profile of the FH12 PHB, potentially influenced by residual inhibitors from the DWG hydrolysate (2.36–6.95 mg/g). The robust detection of principal signals (C=O at ≈180 ppm, —CH_2_— at ≈63 ppm, and —CH— at 71–75 ppm) confirms the authentic PHB structure. Such variations are typical across different experimental conditions, extraction methods, and polymer purity levels (Mohapatra et al. 2017; Sirohi et al. 2021).

**FIGURE 1:**
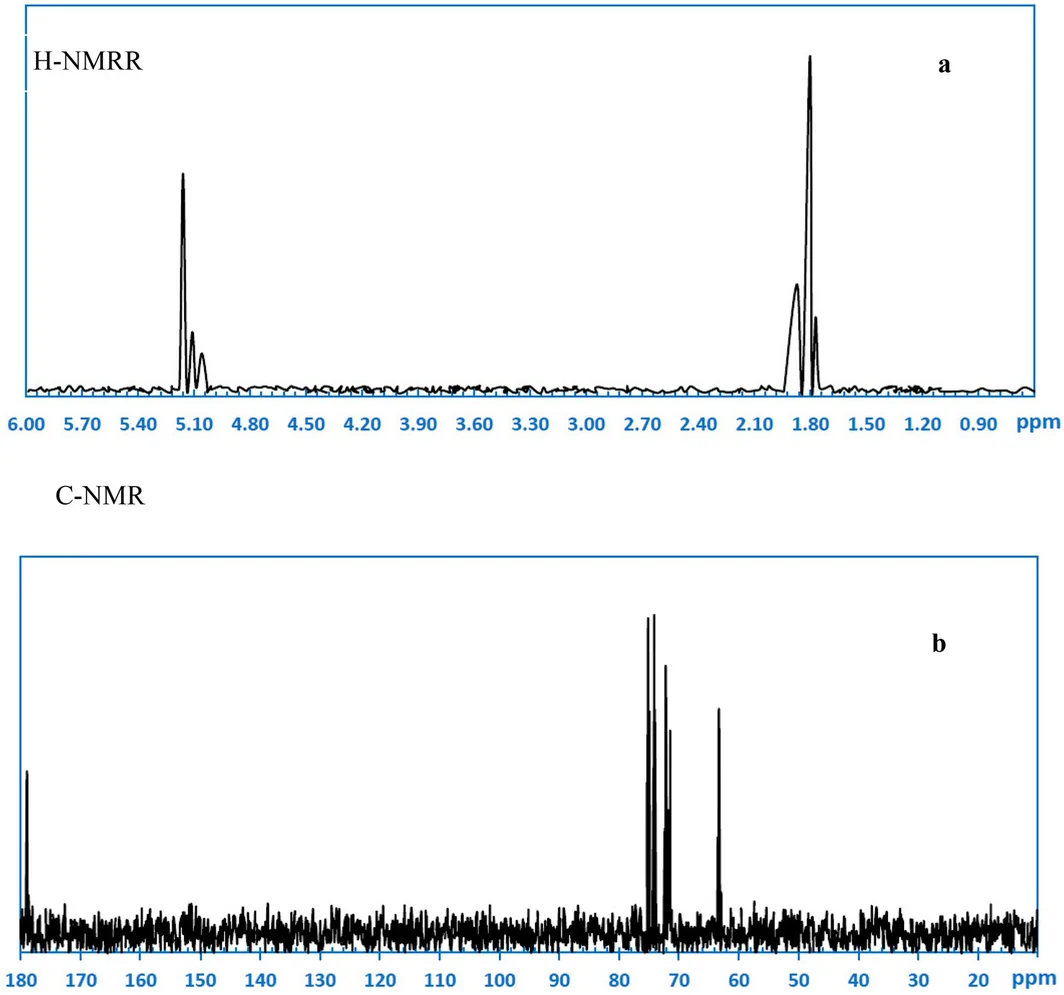
^1^ H−NMR (a) and ^13^ C−NMR (b) spectra of PHB extracted from the FH12 fermentation treatment ($1.85\pm 0.42\;g\;L^{-1}$).

#### 3.4.2 FTIR Analysis

The PHB sample obtained from FH12, which showed the highest PHB production (1.85 ± 0.42 g/L) was selected for FTIR analysis (Figure 2). A broad absorption band at 3468 cm^−1^ was attributed to O—H stretching vibrations, which may be associated with hydroxyl groups in the PHB structure or with residual moisture, proteins, or saccharides from the DWG hydrolysate fermentation medium (Etxabide et al. 2022; Sirohi 2021; Sirohi et al. 2021). Distinct C—H stretching bands were observed at 3013 and 2951 cm^−1^, corresponding to methylene (—CH_2_—) and methyl (—CH_3_) groups, respectively, which are characteristic of PHB (Etxabide et al. 2022; Sirohi 2021). A sharp peak at 1851 cm^−1^ may be attributed to a carbonyl overtone or combination band, or possibly to short‐chain oligomers. The strong absorption band at 1732 cm^−1^, assigned to ester carbonyl (C=O) stretching, confirmed the characteristic aliphatic polyester structure of PHB. A broad band at 1536 cm^−1^ was assigned to —C—CH and —C—CH3 vibrations (Etxabide et al. 2022), although this peak may also indicate the presence of residual amino acids or phenolic compounds from the HDWG4 medium. The FTIR spectrum of the extracted PHB closely resembled that of commercial PHB standards, confirming the authenticity of the polymer structure despite minor variations that may be due to incomplete purification. Characteristic C—O—C stretching vibrations were observed at 1174 and 1009 cm^−1^ while the band at 921 cm^−1^ was assigned to C—O and C—C vibrations associated with the crystalline regions of PHB (Etxabide et al. 2022). Previous studies have reported crystallinity values ranging from 50% to 65.2% for DWG‐derived PHB (Sirohi 2021; Sirohi et al. 2021). Despite the possible presence of minor residual fermentation components, the overall FTIR profile confirmed that the polymer produced by FH12 was structurally authentic PHB and may be suitable for biodegradable packaging applications.

**FIGURE 2:**
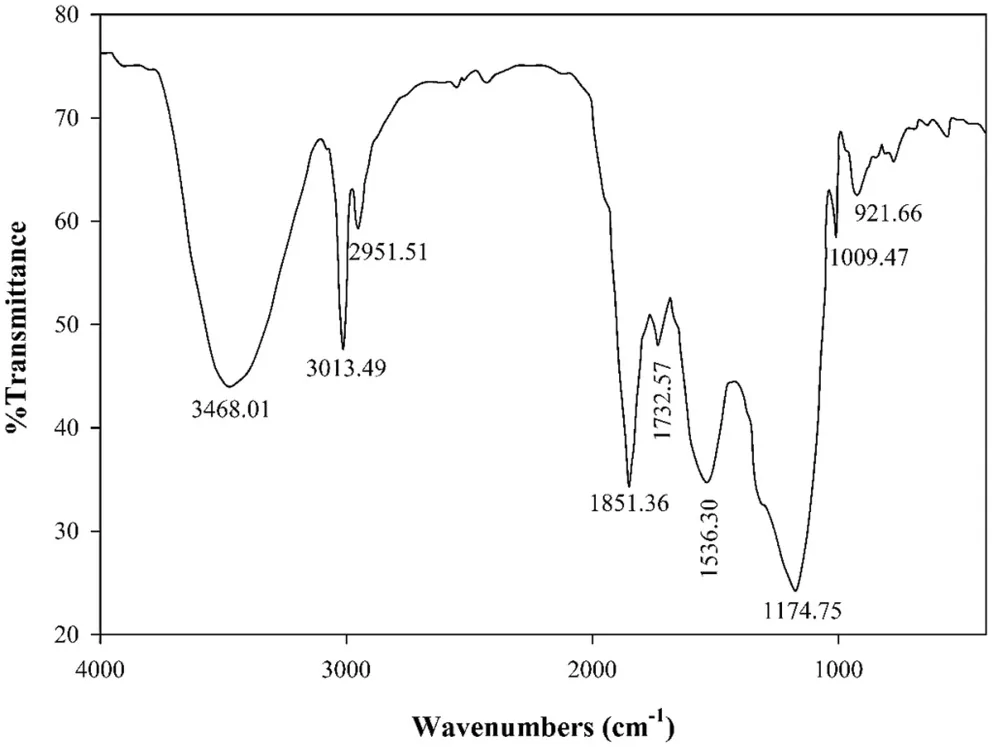
FTIR spectrum of FH12 produced through fermentation.

Characteristic bands were observed in the fingerprint region at 1174.75, 1009.47, and 921.66 cm^−1^. The band at 1174.75 cm^−1^ was mainly assigned to asymmetric C—O—C stretching of the ester groups in PHB. The band at 1009.47 cm^−1^ may be associated with C—O—C and C—O stretching vibrations within the ester‐containing polymer chain, whereas the band at 921.66 cm^−1^ was attributed to C—C and C—O skeletal vibrations associated with ordered PHB chain conformations. The latter bands may be influenced by the crystalline organization of the polymer; however, their assignment should be interpreted together with the XRD results rather than as independent proof of crystallinity. The overall FTIR profile was consistent with the characteristic structure of PHB, although minor residual fermentation components cannot be completely excluded.

#### 3.4.3 Thermal Properties

The thermal degradation behavior of PHB produced by FH12 ($1.85\pm 0.42\;g\;L^{-1}$) was evaluated using thermogravimetric analysis (TGA), and the corresponding thermogram is presented in Figure 3a. A predominantly single‐step mass‐loss event began at approximately 270°C (onset) and progressed through the main degradation region (≈270°C–340°C), indicating rapid PHB decomposition. This behavior is typically attributed to β‐elimination and scission of ester linkages (C[chemistry double bond solid lines]O and C[chemistry single bond solid line]O), leading to the formation of oligomeric products and crotonic acid, accompanied by a marked reduction in molecular weight. The observed profile is consistent with the findings of Etxabide et al. (2022), who reported a similar single‐stage degradation pattern for PHB derived from red grape pomace, characterized by a steep decline between 280°C and 340°C. In contrast, PHB produced from DWG has previously been reported to decompose at 228.6°C, which is substantially lower than the degradation range observed in this study, indicating improved thermal stability of FH12‐derived PHB (Sirohi et al. 2021).

**FIGURE 3:**
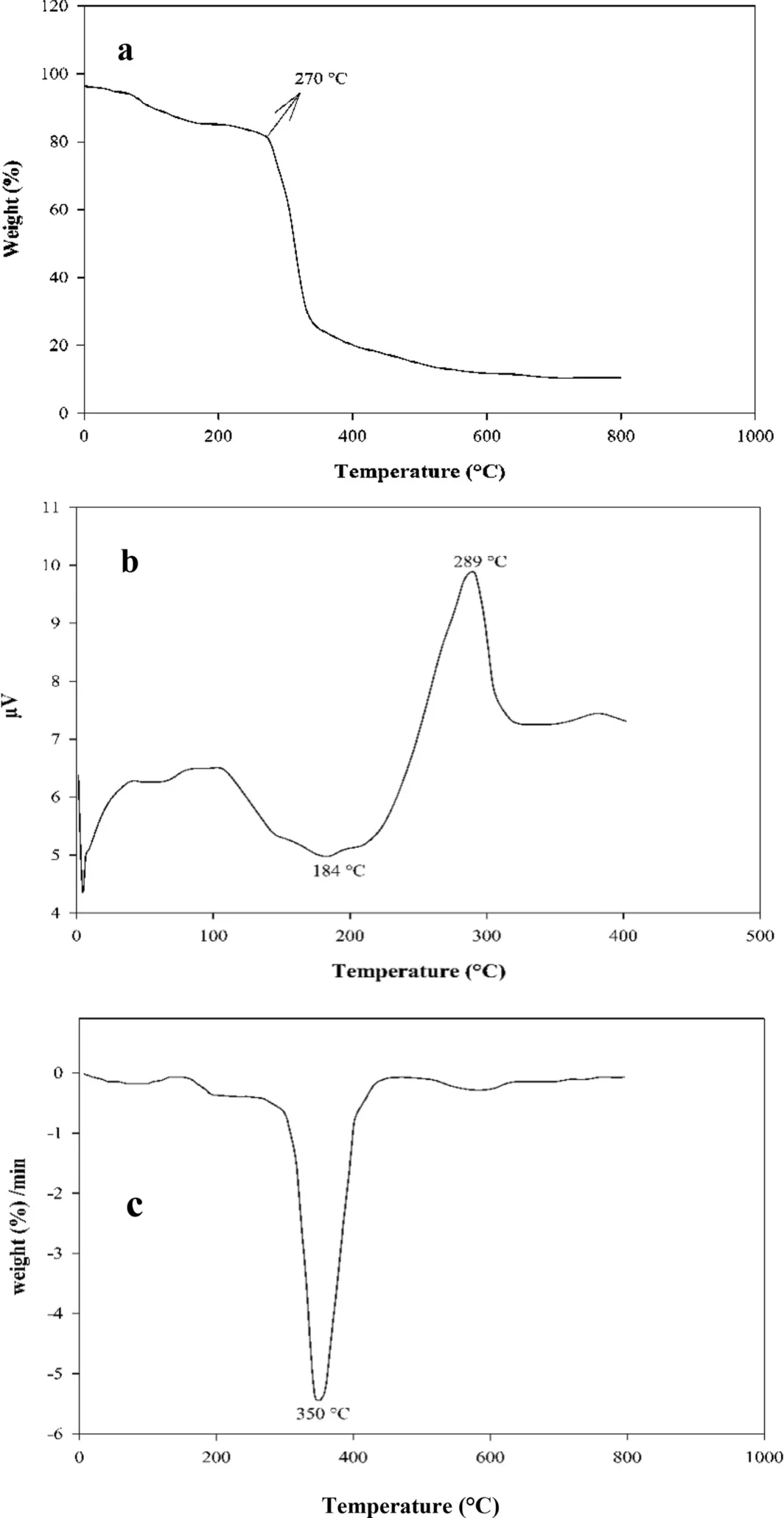
Thermal analysis of PHB produced by FH12 ($1.85\pm 0.42\;g\;L^{-1}$): (a) thermogravimetric analysis (TGA), (b) differential thermal analysis (DTA), and (c) derivative thermogravimetry (DTG).

Differential thermal analysis (DTA) (Figure 3b) revealed an endothermic peak at 184°C corresponding to the melting temperature ($T_{m}$) of PHB, followed by a sharp exothermic peak at 289°C associated with polymer decomposition and structural transitions. The melting temperature of 184°C falls within the typical range reported for PHB produced by various microorganisms (e.g., ~165°C for PHB synthesized by *Alcaligenes latus* using sugar beet juice; Wang et al. 2013), indicating comparable thermal characteristics. The exothermic event near 289°C coincided with the main mass‐loss region observed in the TGA curve and reflects accelerated chain scission and combustion of degradation products. Overall, the thermal stability of FH12‐derived PHB up to approximately 300°C suggests its suitability for processing and applications requiring moderate‐to‐high thermal resistance.

The derivative thermogravimetric (DTG) curve (Figure 3c) further illustrates the rate of mass loss during thermal degradation. FH12 exhibited a maximum degradation rate ($\mathrm{DT}G_{\max}$) at approximately 350°C, with an overall mass loss of about 55%. This degradation peak is notably higher than that typically reported for conventional PHB (≈270°C–300°C) as well as PHB derived from red grape pomace ($\mathrm{DT}G_{\max}\approx 314^{\circ }C$; ∼66% mass loss; Etxabide et al. 2022) and *Bacillus* sp. NII2 grown on DWG ($\mathrm{DT}G_{\max}\approx 300^{\circ }C$; onset $\approx 256^{\circ }C$; Sirohi et al. 2021).

This enhanced thermal stability can be attributed to the high molecular weight, intact macromolecular backbone, and high degree of structural crystallinity achieved through the optimized bioprocessing and mild purification protocol. High‐ordered crystalline domains and the absence of residual pro‐degrading catalytic impurities (such as free metal ions or residual acidic fractions) delay the onset of random chain scission and β‐elimination reactions across the ester backbones. Consequently, the decomposition kinetics are shifted toward higher degradation temperatures under an inert nitrogen atmosphere.

#### 3.4.4 Crystallinity Analysis

The crystalline structure of the biosynthesized PHB produced by the FH12 treatment (with a yield of 1.85 ± 0.42 g/L) was evaluated using X‐ray diffraction (XRD), and the corresponding diffractogram is presented in Figure 4a. The XRD pattern (intensity vs. *2θ*) exhibited two prominent, sharp peaks at *2θ* = 5.8° and 13.51° along with three additional reflection peaks at 21.15°, 26.42°, and 30.60°. The occurrence of these intense and well‐resolved diffraction peaks confirms the highly crystalline nature of the biopolymer synthesized by FH12. The crystalline regions of PHB are formed through the ordered folding and dense packing of linear polymer chains, whereas the disordered, unfolded segments constitute the amorphous matrix. The intense diffraction peak observed at 13.51° corresponds to the (020) and (110) reflections of the orthorhombic PHB crystal lattice, which is in excellent agreement with standard crystallographic assignments for PHB (Sirohi et al. 2021). These findings verify that the polymer produced by FH12 retains the authentic orthorhombic crystalline structure characteristic of high‐quality PHB. For comparison, PHB synthesized from DWG by *Bacillus* sp. NII2 has been reported to display major reflections at 13.45°, 16.89°, and 22.37° (Sirohi et al. 2021). While minor variations in peak positions and intensities can occur due to differences in microbial strains, carbon substrates, and extraction/purification efficiency, the overall diffraction profile of the FH12‐derived polymer confirms it is a highly crystalline, structurally authentic PHB.

**FIGURE 4:**
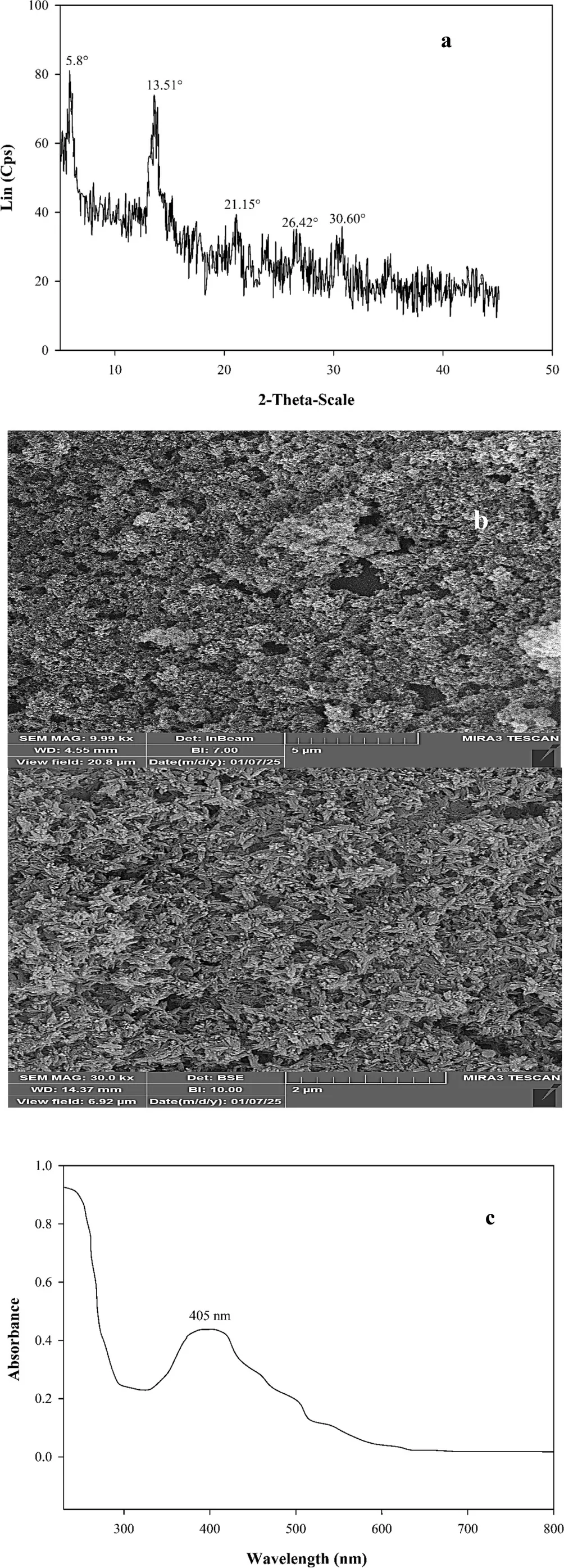
(a) XRD analysis of FH12; (b) SEM analysis of FH12; (c) Optical spectroscopy of FH12.

#### 3.4.5 Morphological Properties

The surface morphology of biosynthesized PHB from the FH12 treatment (1.85 ± 0.42 g/L) was characterized via using SEM analysis (Figure 4b). The micrograph reveals a relatively homogeneous, integrated surface architecture characterized by a rugged, granular texture with non‐uniformly distributed pores and micro‐voids. This continuous phase morphology is characteristic of polyhydroxyalkanoates (PHAs), which typically exhibit a dense, cohesive matrix capable of forming robust films for bioplastic applications (Sirohi et al. 2021). The observed morphology is consistent with previously reported SEM findings for DWG‐derived PHB, confirming the structural integrity and coherent matrix formation of the FH12‐produced polymer (Sirohi et al. 2021).

#### 3.4.6 Optical Spectroscopy

The optical characteristics of PHB recovered from the FH12 fermentation treatment (1.85 ± 0.42 g L^−1^) were evaluated by UV–Vis spectroscopy, and the spectrum is presented in Figure 4c. A high absorbance was observed in the short‐wavelength UV region, followed by a marked decrease between approximately 230 and 329 nm. This behavior indicates attenuation of incident UV radiation by the recovered material. A broad absorbance maximum of approximately 0.43 was observed near 405 nm, within the violet–blue region of the visible spectrum. Because purified PHB is generally weakly absorbing in the visible range, this feature may be associated with residual chromophoric compounds from the DWG hydrolysate or fermentation medium, such as phenolic or other oxidized constituents, rather than being assigned exclusively to the PHB backbone. The absorbance subsequently decreased from approximately 405 to 800 nm and approached a low, nearly constant value above approximately 650 nm. Thus, the spectrum indicates partial UV attenuation and a weak broad absorption in the violet–blue region; however, the potential packaging relevance of the material cannot be confirmed from UV–Vis data alone and would require assessment of purification, color, transparency, and film‐level optical properties.

## 4 Conclusions

This study successfully demonstrated the feasibility of producing poly(3‐hydroxybutyrate) (PHB) from damaged wheat grain (DWG) as a low‐cost, non‐food agro‐industrial waste substrate, thereby providing a promising approach for biomass valorization within a circular bioeconomy framework. The initial physicochemical characterization of DWG confirmed its suitability for biotechnological application due to its appreciable nutritional composition and the presence of heavy metals within safe limits. Optimization of acid hydrolysis conditions showed that treatment with 6% HCl, using a 20% acid‐to‐sample ratio, 20% substrate loading, and a 45 min hydrolysis time, produced the most favorable hydrolysate (HDWG4). This hydrolysate yielded the highest reducing sugar concentration (336.66 mg/g), retained relatively high protein content, and contained only low levels of inhibitory compounds, with acetic and formic acids ranging from 2.36 to 6.95 mg/g. These characteristics indicate that HDWG4 was suitable for subsequent microbial fermentation.

Among the tested bacterial isolates, including *Lactobacillus plantarum* PTCC 1058, *Lactobacillus murinus* PTCC 322, and *Lactobacillus casei* PTCC 1608, the FH12 treatment showed the highest PHB‐producing capability under the optimized fermentation conditions of 30°C, 6% inoculum, and 96 h cultivation time. Under these conditions, FH12 produced 1.85 ± 0.42 g/L PHB and achieved 18.5% accumulation relative to dry cell weight (DCW). Although this value is lower than that typically reported for conventional industrial PHB producers, it is significant considering the use of a complex lignocellulosic/agro‐waste‐derived hydrolysate and *Lactobacillus* as a non‐traditional PHB‐producing bacterium. Comprehensive characterization confirmed the successful biosynthesis and quality of the extracted polymer. FTIR and NMR analyses verified the characteristic chemical structure of PHB. XRD analysis demonstrated the semi‐crystalline nature of the polymer and confirmed the presence of the typical orthorhombic crystal structure of PHB. SEM observations revealed a relatively homogeneous and cohesive surface morphology with non‐uniformly distributed pores, indicating the formation of a structured polymer matrix. In addition, UV–Vis analysis showed strong absorption in the 230–329 nm range, suggesting favorable UV‐blocking properties that may be advantageous for packaging applications. Thermal characterization by TGA/DTG/DTA further indicated that the produced PHB exhibited high thermal stability, with major degradation occurring at elevated temperatures compared with values reported in previous studies. Overall, the findings of this study confirm that damaged wheat grain can be effectively converted into value‐added biopolymer through acid hydrolysis followed by microbial fermentation using *Lactobacillus* species under treatment FH12. The structural authenticity, crystallinity, thermal stability, surface morphology, and UV‐protective properties of the produced PHB highlight its potential as a biodegradable material for sustainable packaging applications. This study is novel in demonstrating the conversion of damaged wheat grain into PHB through optimized acid hydrolysis coupled with a *Lactobacillus* consortium, offering a sustainable bioprocess for the valorization of underutilized agro‐waste.

Future work should focus on improving PHB yield and productivity through hydrolysate detoxification, strain enhancement, and process optimization to further strengthen its industrial applicability.

## Author Contributions

**Zohreh Boorboormoradi:** formal analysis, writing – original draft. **Orang Eyvazzade:** writing – review and editing. **Ladan Rashidi:** supervision, writing – review and editing, methodology. **Leila Nateghi:** writing – review and editing.

## Funding

The authors have nothing to report.

## Conflicts of Interest

The authors declare no conflicts of interest.

## Data Availability

The current study is available from the corresponding author upon reasonable request.
